# Antimicrobial Activity of the Peptide LfcinB15 against *Candida albicans*

**DOI:** 10.3390/jof7070519

**Published:** 2021-06-29

**Authors:** Che-Kang Chang, Mou-Chieh Kao, Chung-Yu Lan

**Affiliations:** 1 Institute of Molecular and Cellular Biology, National Tsing Hua University, Hsinchu 30013, Taiwan; steve2202543@gapp.nthu.edu.tw; 2 Institute of Molecular Medicine, National Tsing Hua University, Hsinchu 30013, Taiwan; 3 Department of Life Science, National Tsing Hua University, Hsinchu 30013, Taiwan

**Keywords:** *C. albicans*, antimicrobial peptide, LfcinB15

## Abstract

Lactoferricin (Lfcin) is an amphipathic, cationic peptide derived from proteolytic cleavage of the N-lobe of lactoferrin (Lf). Lfcin and its derivatives possess broad-spectrum antibacterial and antifungal activities. However, unlike their antibacterial functions, the modes of action of Lfcin and its derivatives against pathogenic fungi are less well understood. In this study, the mechanisms of LfcinB15, a derivative of bovine Lfcin, against *Candida albicans* were, therefore, extensively investigated. LfcinB15 exhibited inhibitory activity against planktonic cells, biofilm cells, and clinical isolates of *C. albicans* and non-*albicans Candida* species. We further demonstrated that LfcinB15 is localized on the cell surface and vacuoles of *C. albicans* cells. Moreover, LfcinB15 uses several different methods to kill *C. albicans*, including disturbing the cell membrane, inducing reactive oxygen species (ROS) generation, and causing mitochondrial dysfunction. Finally, the Hog1 and Mkc1 mitogen-activated protein kinases were both activated in *C. albicans* cells in response to LfcinB15. These findings help us to obtain more insight into the complex mechanisms used by LfcinB15 and other Lfcin-derived peptides to fight fungal pathogens.

## 1. Introduction

*Candida albicans* is a commensal yeast inhabiting the skin and various mucosal surfaces of the human body. However, *C. albicans* is also an opportunistic pathogen, particularly for immunocompromised patients, and can cause a range of diseases such as superficial infections and invasive candidiasis, with high mortality rates [1,2,3]. Moreover, the emergence of antifungal resistance in *C. albicans* has become a serious threat in the clinical setting [4].

Antimicrobial peptides (AMPs) are important components of the human innate immune system and are considered promising candidates for developing new antifungals for the treatment of drug-resistant infections [5,6]. AMPs generally consist of 10 to 100 amino acid residues and are divided into four classes based on their secondary structures, including α-helical, β-sheet, extended conformation and β-hairpin or loop peptides [7,8]. Moreover, antimicrobial activities are diverse among different types of AMPs. Some AMPs interact directly with the negatively charged cytoplasmic membrane of microbes, leading to disruption of the membrane [9]. Alternatively, some AMPs translocate into the cytosol to interfere with distinct bioprocesses such as DNA replication and protein synthesis [10]. For example, Tur1A and histatin 5 (Hst 5) translocate intracellularly and exert their antimicrobial activity without membrane disruption [11,12].

Lactoferrin (Lf) is a glycoprotein belonging to the transferrin family of proteins, which are able to bind and transfer iron(III) ions [13]. Human Lf consists of two homologous N- and C-terminal globular lobes [14] and is found in specific granules of neutrophils, epithelia and body secretions such as milk and saliva [14]. Importantly, human Lf possesses antimicrobial, antioxidant, antitumor, antiallergic, anti-inflammatory and immunoregulatory activities, and iron sequestration is associated with its ability to inhibit bacterial growth [15]. In addition to human Lf, bovine Lf is another well-studied form of lactoferrin, sharing high sequence homology with human Lf [14]. Moreover, lactoferricin B (LfcinB) is a 25-amino-acid peptide corresponding to residues 17–41 of bovine Lf generated by acid-pepsin digestion. LfcinB not only has antiviral, antitumor and immunological properties [16] but also notably exhibits even more potent antibacterial activity than intact bovine and human Lf [17,18,19,20]. In addition, LfcinB has great efficacy against a broad spectrum of fungi, including species of *Candida*, *Cryptococcus*, *Aspergillus* and *Penicillium* [21].

Many derivatives of bovine LfcinB have been generated and characterized. For example, the peptide consisting of the 1–10, 4–12 or 2–21 residues of LfcinB (corresponding to positions 17–26, 20–28 or 18–37 of bovine Lf, respectively), exhibiting potent candidacidal activity [21]. Moreover, LfcinB15 is a 15-residue truncated fragment corresponding to residues 1–15 of LfcinB (i.e., positions 17–31 of bovine Lf) and still maintains nearly the same effective antibacterial activity as its parental LfcinB [22]. In addition, LfcinB15 showed low hemolytic activity (less than 8% lysis) with the concentration ranging from 4 to 256 µg/mL [22]. However, the antifungal activity and the detailed fungal killing mechanism of LfcinB15 have not yet been characterized.

In this study, we investigated LfcinB15 to gain a better understanding of the activity and modes of action of LfcinB-derived peptides against *C. albicans*. First, LfcinB15 exhibited antifungal activity against planktonic cells, biofilm cells and clinical isolates of *C. albicans* and non-*albicans Candida* species. Second, LfcinB15 exerted anti-*C. albicans* activity through multiple modes of action, including membrane disruption, ROS accumulation and mitochondrial dysfunction. Finally, the *C. albicans* stress response protein kinases Hog1 and Mkc1 were activated by LfcinB15, and the *HOG1*-deleted (*hog1*Δ) mutant was significantly resistant to LfcinB15. Together, these findings provide insights into the complex mechanisms of LfcinB15 and LfcinB-derived peptides against fungal pathogens.

## 2. Materials and Methods

### 2.1. C. albicans Strains, Media and Growth Conditions

The *C. albicans* SC5314 strain was used throughout this study unless stated otherwise. The *C. albicans* mutant strains that are deficient in mitochondrial electron transport chain (ETC) and their parental strain SN250 [23] were used as indicated. For the determination of minimal inhibitory concentration (MIC), different *C. albicans* and non-*albicans Candida* clinical isolates were also used [24]. Cells were maintained at −80 °C and plated on YPD agar (1% yeast extract, 2% peptone, 2% glucose and 1.5% agar) before each experiment. A single colony was inoculated into YPD broth and grown at 30 °C overnight (~16 h) with shaking at 180 rpm. The overnight culture was subcultured in YPD (with an initial concentration ~3 × 10^6^ CFU/mL) and further grown at 30 °C to the exponential phase.

### 2.2. Peptides and Reagents

LfcinB15 (FKCRRWQWRMKKLGA-NH_2_) and fluorescein isothiocyanate (FITC)-conjugated LfcinB15 (FITC-FKCRRWQWRMKKLGA-NH_2_) were synthesized by Mission Biotech, Inc. (Taipei, Taiwan). The purity of the peptides was determined by reversed-phase high-performance liquid chromatography (HPLC, Shimadzu, Kyoto, Japan) and mass spectrometry to be >95% pure. The stock solution (5 mg/mL) was prepared by dissolving LfcinB15 or FITC-LfcinB15 in sterile double-distilled water. All reagents were purchased from Sigma-Aldrich (St. Louis, MO, USA) unless stated otherwise.

### 2.3. Cell Susceptibility to LfcinB15

Cell susceptibility to LfcinB15 was examined by colony forming unit (CFU) counting and propidium iodide (PI) staining. Briefly, *C. albicans* cells were suspended to a final concentration of ~3 × 10^8^ CFU/mL with 12.5 mM sodium acetate in a 1.5 mL microtube and 100 µL of *C. albicans* cell suspension was incubated with or without different concentrations of LfcinB15 at 37 °C for 1 h. Subsequently, cells were 10-fold serially diluted, and grown on YPD agar plates and incubated at 30 °C overnight, and the number of CFUs was counted.

For PI staining, cells (~3 × 10^8^ CFU/mL in 12.5 mM sodium acetate) were treated with or without different concentrations of LfcinB15 at 37 °C for 1 h, harvested by centrifugation, washed with phosphate buffered saline (PBS), resuspended in a PI staining solution (6 µg/mL PI in PBS), and incubated at 37 °C for 5 min. PI-positive cells were measured using an Accuri C6 flow cytometer (BD Biosciences, San Jose, CA, USA), and data were processed using BD Accuri C6 software.

### 2.4. Minimal Inhibitory Concentration (MIC) of LfcinB15

The MIC of clinically isolated *Candida* strains to LfcinB15 was determined by the broth dilution method as described in the Clinical and Laboratory Standards Institute document M27-A3 [25], with some modifications, as previously described [26]. Briefly, cells were diluted to a final concentration of 2 × 10^4^ CFU/mL with low salt LYM broth [26]. Two hundred microliters of cell suspension were added to each well of a 96-well microplate and incubated with the indicated concentration of LfcinB15 at 37 °C for 48 h. The absorbance at 595 nm was then measured using a Bio-Rad iMarkTM microplate reader (Bio-Rad, Hercules, CA, USA). The MIC was defined as the concentration of LfcinB15 that caused a complete inhibition in growth relative to the LfcinB15-free control. The experiments were repeated at least three times.

### 2.5. Assessment of Biofilm Formation

The XTT reduction assay was performed to measure biofilm formation, as previously described [27]. After overnight growth, *C. albicans* cells were suspended to a final concentration of 3 × 10^5^ CFU/mL in RPMI 1640 medium (Thermo Fisher, Waltham, MA, USA) with 3.453% MOPS and 1.8% glucose. Cells were then added to each well of a 96-well polystyrene microplate and incubated for 24 h at 37 °C with 5% CO_2_. Subsequently, the adherent cells in the biofilm were washed and incubated with or without different concentrations of LfcinB15 (in 12.5 mM sodium acetate) at 37 °C for 1 h. The supernatant in each well was carefully removed, and 300 μL of 2,3-bis-(2-methoxy-4-nitro-5-sulfophenyl)-2H-tetrazolium-5-carboxanilide (XTT) with 0.5 μM menadione in PBS was added to the biofilm cells and incubated at 37 °C for 15 min. The XTT reduction was measured at 490 nm using a Bio-RAD iMarkTM microplate reader.

For the examination of the *C. albicans* biofilm structure, biofilms were formed on Thermanox polystyrene coverslips (Thermo Fisher) and examined by SEM, as previously described [27]. Briefly, biofilm cells on coverslips were treated with or without LfcinB15 (100 and 200 µg/mL), incubated at 37 °C for 1 h, and washed twice with PBS. The coverslips were then fixed with 3.75% formaldehyde (in PBS) at room temperature (RT) for 40 min, followed by incubation with 1% osmium tetroxide (in PBS; Electron Microscopy Sciences, Hatfield, PA, USA) for 5 min and dehydration with serial ethanol solutions [27]. The samples were dried in a 60 °C oven. Finally, the biofilms were examined, and micrographs were collected using SEM (Hitachi, S-4700, Type II).

### 2.6. Fluorescence Microscopy

To determine the subcellular localization of LfcinB15, cells were simultaneously stained with FITC-LfcinB15 and calcofluor white (CFW), 7-amino-4-chloromethylcoumarin (CellTracker Blue CMAC, PerkinElmer, Carlsbad, CA, USA), MitoTracker red (MTR; Invitrogen, Eugene, OR, USA) or 4′,6-diamidino-2-phenylindole (DAPI). Briefly, *C. albicans* cells were suspended to a final concentration of 3 × 10^8^ CFU/mL with 12.5 mM sodium acetate and incubated with 6.25 μg/mL LfcinB15 at 37 °C for 15 min. For organelle or cell wall staining, cells were independently incubated with 1 μg/mL CFW, 100 μM CMAC, 75 nM MTR, or 1 μg/mL DAPI. The samples were subsequently examined using a CLSM system (Zeiss LSM800 with Airyscan; Carl Zeiss, Jena, Germany) at 100× magnification. The images were processed using ZEISS ZEN software version 1.1.2.0.

### 2.7. Liposome Preparation and Determination of Membrane Disturbance by Measuring Calcein Leakage

Small unilamellar liposome vesicles (SUVs; 100 nm diameter) were prepared, as previously described [28], with some modifications. Briefly, phosphatidylcholine (PC), phosphatidylethanolamine (PE) and phosphatidylinositol were mixed with ergosterol at a ratio of 5:4:1:2 (*w*/*w*/*w*/*w*) in chloroform. PC, PE and phosphatidylinositol were purchased from Avanti Polar Lipids (Alabaster, AL, USA). The mixture was then dried using a stream of nitrogen gas, and the lipid film was vigorously mixed with liposome buffer (70 mM calcein, 10 mM Tris, 75 mM NaCl, and 0.1 mM EDTA, pH 7.4) for 30 min. Subsequently, the solution was repeatedly frozen and thawed with a −80 °C refrigerator for 10 cycles and extruded through a 100 nm polycarbonate filter 5 times. The SUVs loaded with calcein were purified with a Sephadex G50 gel filtration column.

To detect the release of calcein, freshly prepared SUVs were treated with or without various concentrations of LfcinB15 for 5 min at RT in a reaction buffer (10 mM Tris, 150 mM NaCl, and 0.1 mM EDTA, pH 7.4). SUVs treated with 0.1% Triton X-100 were used as a positive control. The fluorescence of calcein release was measured using a Victor3™ Plate Reader (PerkinElmer, Santa Clara, CA, USA) at excitation and emission wavelengths of 495 and 515 nm, respectively. The percentage of calcein release was calculated as follows: calcein release = (F − F_0_)/(F_t_ − F_0_) × 100%, where F is the fluorescence intensity after LfcinB15 treatment, F_t_ is the fluorescence intensity with Triton X-100 treatment, and F_0_ is the fluorescence intensity of the untreated control SUVs.

### 2.8. Measurement of Intracellular ROS

Intracellular ROS levels were measured using dihydroethidium (DHE), 2′,7′-dichlorodihydrofluorescein diacetate (H_2_DCFDA), and MitoSOX red (Invitrogen), as previously described [29]. Briefly, cells (3 × 10^8^ CFU/mL) were treated with or without different concentrations of LfcinB15 for 1 h. The cells were collected, washed with PBS, resuspended in PBS containing either 20 μg/mL H_2_DCFDA or 5 μM MitoSOX Red, and incubated at 30 °C for 30 and 20 min, respectively. For DHE staining, cells were resuspended in PBS containing 20 μM DHE and incubated at 37 °C for 5 min. The fluorescence intensity was measured using an AccuriTM C6 flow cytometer (BD Biosciences), and the mean fluorescence intensity was calculated using BD Accuri C6 software.

### 2.9. Cell Rescue Assay Using ROS Scavengers

To determine the relationship between ROS generation and *C. albicans* killing by LfcinB15, the ROS scavengers NAC and glutathione were used. Briefly, 3 × 10^8^ CFU/mL cells were suspended in 12.5 mM sodium acetate and incubated at 37 °C with either 60 mM N-acetyl cysteine (NAC) or 15 mM glutathione for 30 min, respectively. Cells were harvested, washed with 12.5 mM sodium acetate, treated with or without different concentrations of LfcinB15, and incubated at 37 °C for 1 h. Cells were then plated on YPD agar plates and incubated overnight at 30 °C, and the number of CFUs from cells treated with LfcinB15 was counted and normalized to that of the untreated control. The results were reported as percentages.

### 2.10. Protein Extraction and Western Blotting

Protein extraction and western blotting were performed as previously described [30]. An anti-phospho-p38 (Thr180/Tyr182) monoclonal antibody (#9211, Cell Signaling Technology, Danvers, MA, USA) and Hog1 (y-215) antibody (sc-9079, Santa Cruz Biotechnology, Inc., Dallas, TX, USA) were used to detect phospho-Hog1 and total Hog1, respectively. Moreover, an anti-p-P44/42 MAPK antibody (4270S, Cell Signaling Technology) was used for the detection of both phosphor-Mkc1 and phosphor-Cek1, and a GAPDH antibody (GTX100118, GeneTex, Hsinchu, Taiwan) was used for GAPDH. Horseradish peroxidase (HRP)-conjugated goat anti-rabbit IgG (GTX213110, GeneTex) was used as the secondary antibody. The blot was visualized using the Western Lightning Plus-ECL Enhanced Chemiluminescence Substrate kit (PerkinElmer) and an ImageQuant LAS 4000 Biomolecular Imager (GE Healthcare Life Science, Marlborough, MA, USA).

### 2.11. Measurement of the Mitochondrial Membrane Potential

The mitochondrial membrane potential was measured by staining cells with rhodamine 123, as previously described [31], and JC-1, according to the manufacturer’s instructions (Thermo Fisher) but with some modifications. For rhodamine 123 staining, cells (3 × 10^8^ CFU/mL) were treated with or without different concentrations of LfcinB15 for 1 h, harvested by centrifugation, resuspended with 25 μM rhodamine 123 (in 50 mM sodium citrate) and incubated at 30 °C for 10 min. For JC-1 staining, cells were harvested, resuspended with 2 µM JC-1 (in PBS) and incubated at 37 °C for 15 min. After staining, cells were washed three times with PBS, and the fluorescence intensity was measured using an AccuriTM C6 flow cytometer (BD Biosciences). Cells without AMP treatment were used as negative controls, whereas cells treated with 15 mM hydrogen peroxide (H_2_O_2_) for 1 h were used as positive controls.

### 2.12. Measurement of Intracellular and Extracellular ATP Levels

The ATP level was determined with a bioluminescence assay using an ATP Determination Kit (A22066, Invitrogen). Briefly, *C. albicans* cells were diluted to a final concentration of 3 × 10^8^ CFU/mL with 12.5 mM sodium acetate, incubated with or without different concentrations of LfcinB15 at 37 °C for 1 h, and collected by centrifugation. The supernatant was kept on ice before use for measuring the extracellular ATP level, while the cell pellets were broken, as previously described [30], for measuring intracellular ATP.

For ATP measurement, the ATP Determination Kit was used according to the manufacturer’s instructions (Invitrogen). Briefly, 1× reaction buffer containing 0.5 mM D-luciferin, 1.25 μg/mL firefly luciferase, and 1 mM DTT was freshly prepared before each experiment, and 10 µL of the supernatant or cell lysate was mixed with 90 µL of the reaction buffer. Then, the mixture was placed into each well of a black 96-well microplate, and luminescence was read using a Victor3™ Plate Reader (PerkinElmer) at 560 nm. A standard curve of increasing ATP concentrations (0, 0.01, 0.1, 1 μM) was created. Signals represented at least three independent experiments were obtained and the ATP concentration of each sample was calculated from the standard curve.

### 2.13. Oxygen Consumption Rate (OCR) Measurement Using High-Resolution Respirometry

The OCR was measured using an Oxygraph-O2k system (Oroboros Instruments, Innsbruck, Austria). Briefly, *C. albicans* cells were diluted to a final concentration of 3 × 10^8^ CFU/mL with 12.5 mM sodium acetate and incubated, with or without 12.5 μg/mL LfcinB15, at 37 °C for 1 h. After incubation, cells were diluted again with PBS to a final concentration of 6 × 10^6^ CFU/mL. The Oroboros Oxygraph-O2k system was first air calibrated with PBS following the manufacturer’s instructions. Then, 3 mL of each sample was loaded into the O2 k chambers, and the OCR was monitored until the signal reached a plateau. For CCCP titration, cells were slowly titrated with 1.5 µL of stock CCCP solution (10 mM in dimethyl sulfoxide or DMSO) 10–20 times until the OCR stopped increasing. Subsequently, cells from each sample were grown on YPD agar plates and incubated at 30 °C overnight, and the number of CFUs was counted. The OCR was calculated as follows: OCR per million CFUs = average OCR reading in the plateau (pmol/s × mL)/the number of millions of CFUs, where s is seconds.

### 2.14. Determination of the Association between LfcinB15 and Mitochondrial Respiration

Susceptibility to rotenone, sodium azide (NaN_3_) and carbonyl cyanide m-chlorophenylhydrazone (CCCP) was assessed to correlate LfcinB15 killing with mitochondrial respiration. For the experiments with rotenone treatment, cells (3 × 10^8^ CFU/mL) were suspended in 12.5 mM sodium acetate, treated with 0.31 mM rotenone, and incubated at 37 °C for 30 min. Cells were harvested, washed with 12.5 mM sodium acetate, and incubated with or without different concentrations of LfcinB15 at 37 °C for 1 h. For the treatment with NaN_3_, cells were harvested, washed with 12.5 mM sodium acetate, and incubated with 5 mM NaN_3_ at 37 °C for 10 min. The NaN_3_-treated cells were further treated with or without different concentrations of LfcinB15 and incubated at 37 °C for 1 h. For the treatment with CCCP, cells were coincubated with or without different concentrations of LfcinB15 and 50 μM CCCP at 37 °C for 1 h. In all the experiments, cells were then plated on YPD agar plates and incubated overnight at 30 °C. The number of CFUs was counted and normalized to that of the control cells. The results are reported as percentages.

The susceptibility of the *C. albicans* mutants that are deficient in mitochondrial ETC (*orf19.4758*, *orf19.7590*, *mci4*, *orf19.1710*, and *cox4*) and *ypt72* genes to LfcinB15 were assessed by CFU counting as described above.

### 2.15. Statistical Analysis

The two-tailed Student’s *t*-test was applied to assess significant differences between samples. Statistical significance was indicated with a *p*-value < 0.05.

## 3. Results

### 3.1. LfcinB15 Exerts Anti-Candida Activity against Both Planktonic and Biofilm Cells

**Figure 1:**
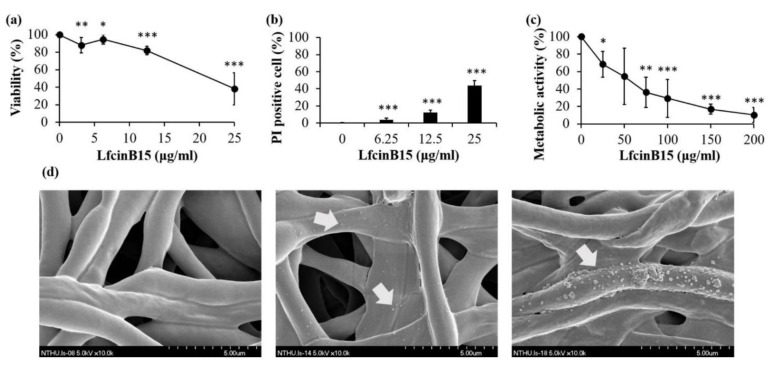
LfcinB15 has anti-*Candida* activity. (**a**) Killing of *C. albicans* by LfcinB15 was determined by counting the number of CFUs and expressed as the percentage of viable cells. The results are presented as the mean ± standard deviation (SD) of three independent experiments; * *p* < 0.05; ** *p* < 0.01; *** *p* < 0.001. (**b**) Killing of *C. albicans* by LfcinB15 was detected by PI staining, and the dead (PI-positive) cells were quantified by flow cytometry and reported as a percentage. The results are presented as the mean ± SD of three independent experiments; *** *p* < 0.001. (**c**) Reduction in metabolic activity of *C. albicans* biofilms after LfcinB15 treatment. The results are reported as percentages, and error bars represent the SD from the mean of three independent experiments. * *p* < 0.05; ** *p* < 0.01; *** *p* < 0.001. (**d**) The morphology of biofilm cells treated without (left) and with 100 μg/mL (middle) and 200 μg/mL (right) LfcinB15 was examined by SEM at a magnification of ×10,000. Arrows indicate the rough appearance of protuberances.

To determine the ability of LfcinB15 to kill *C. albicans*, CFU counting and PI staining were performed. The viability of cells treated with LfcinB15 was compared to that of untreated control cells. The results indicated that cell viability was decreased as the LfcinB15 concentration increased (Figure 1a). Cell viability was ~80% and ~40% after treatment with 12.5 μg/mL and 25 μg/mL LfcinB15, respectively. Moreover, PI is a cell-impermeable intercalating dye that stains nucleic acids of dead cells and is used to distinguish between living and dead cells. Cell death (PI-positive cells) was induced with increasing concentrations of LfcinB15 (Figure 1b).

Biofilms are important for *C. albicans* survival and dispersion for colonization of new niches within the human host. Moreover, biofilm cells exhibit resistance to currently available antifungals and are closely related to infection [32]. To assess the efficacy of LfcinB15 against biofilm cells, biofilms were formed and incubated with different concentrations of LfcinB15, followed by measuring the metabolic activity of *C. albicans* biofilms using the XTT reduction assay [27]. The results indicated that increasing concentrations of LfcinB15 significantly reduced biofilm cells compared to the control without LfcinB15 treatment (Figure 1c). To further examine the effect of LfcinB15 on biofilm cells, the structure of the formed biofilms was also examined by scanning electron microscopy (SEM). As indicated, the surfaces of cells without LfcinB15 treatment were smooth, whereas the surfaces of treated cells showed a rough appearance with protuberances (Figure 1d). Overall, LfcinB15 was effective against both planktonic and biofilm *C. albicans* cells in a dose-dependent manner, and the structure of the biofilm was altered by LfcinB15.

### 3.2. LfcinB15 Also Exerts Antifungal Activity against Clinical Isolates of Candida Species

To further verify the efficacy of LfcinB15, the susceptibility of fourteen *C. albicans* and non-*albicans Candida* clinical isolates to the peptide was determined by the broth dilution method [25]. The results showed that the minimal inhibitory concentration (MIC) of LfcinB15 in ten of the clinical isolates was similar to or lower than that in the reference *C. albicans* SC5314 strain (Table 1). Notably, among these ten isolates, YH050072, YH050075, YLO86, YH050007, YH050013, and YH050114 are fluconazole resistant [24]. Moreover, *C. glabrata* isolates were much more resistant to LfcinB15 than *C. albicans* and other non-*albicans Candida* isolates. This finding is consistent with previous studies in which clinical isolates of *C. glabrata* were found to be somehow more resistant than other *Candida* species to different AMPs [33,34,35].

### 3.3. LfcinB15 Disturbs Membrane Integrity and Enters C. albicans Cells

**Figure 2:**
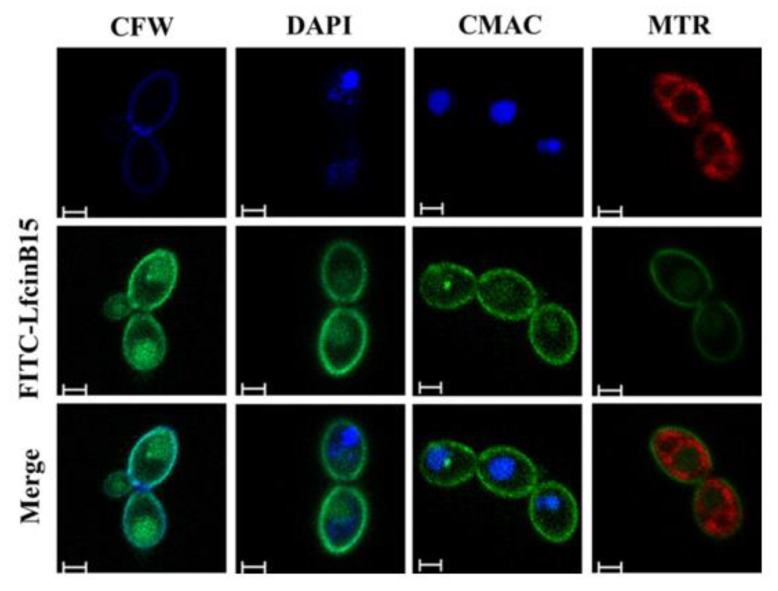
FITC-labeled LfcinB15 is mainly localized to the *C. albicans* cell surface and vacuole. Fluorescence images show different subcellular localization of FITC-LfcinB15 by CLSM at a magnification of ×100. CFW: calcofluor white; DAPI: 4′,6-diamidino-2-phenylindole; CMAC: CellTracker Blue CMAC (7-amino-4-chloromethylcoumarin) dye; MTR: MitoTracker red. Scar bar: 2 µm.

To reveal the mechanisms of action of LfcinB15, FITC-conjugated LfcinB15 was used to determine the cellular localization of the peptide. *C. albicans* was coincubated with FITC-LfcinB15 and CFW, a fluorescent cell wall stain, followed by examining cells with confocal laser scanning microscopy (CLSM). Figure 2 shows that FITC-LfcinB15 colocalized with CFW, suggesting that the peptide interacts with the cell surface of *C. albicans*. Moreover, to determine whether the peptide can further gain access into cells, *C. albicans* cells were coincubated with FITC-LfcinB15 and various organelle-specific dyes, including DAPI, CMAC and MTR, which are used for staining the nucleus, vacuole and mitochondria, respectively. The images indicated that FITC-LfcinB15 colocalized with CMAC but not DAPI or MTR (Figure 2). Therefore, the peptide is not only associated with the cell surface but can also enter the cell to accumulate in vacuoles.

**Figure 3:**
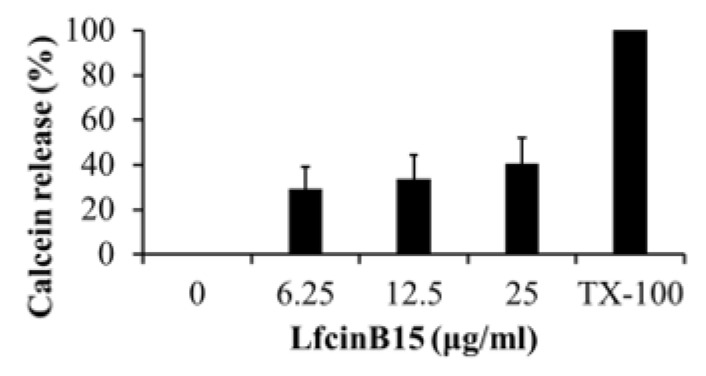
LfcinB15 disturbs the membrane integrity. The release of calcein from SUVs (PC:PE: phosphatidylinositol:ergosterol = 5:4:1:2(*w*/*w*/*w*/*w*)) was detected after treatment with different concentrations of LfcinB15 for 5 min. The release of calcein was expressed as a percentage. Calcein release from the SUVs treated with 0.1% Triton X-100 was defined as 100% release. TX-100: Triton X-100. The error bars represent SD from the mean of three independent experiments.

AMPs generally function in either membrane or nonmembrane targeting [36]. Because LfcinB15 associates with the cell surface (Figure 2), we were thus interested in further determining whether LfcinB15 has membrane-disturbing ability, which was assessed by a SUV-calcein leakage assay. These SUVs mimicking the plasma membrane of *C. albicans* were prepared as previously described [28]. In this assay, calcein-loaded SUVs were treated with various concentrations of LfcinB15 for 5 min, and the leakage of calcein was detected using a fluorescence spectrometer. The calcein released from SUVs treated with 0.1% Triton X-100 and without LfcinB15 treatment was used as a positive and negative control, respectively. As shown in Figure 3, different concentrations of LfcinB15 induced leakage of calcein compared to the controls, suggesting that LfcinB15 can disturb membrane integrity.

### 3.4. LfcinB15-Induced ROS Production Is Related to Its Candidacidal Activity

**Figure 4:**
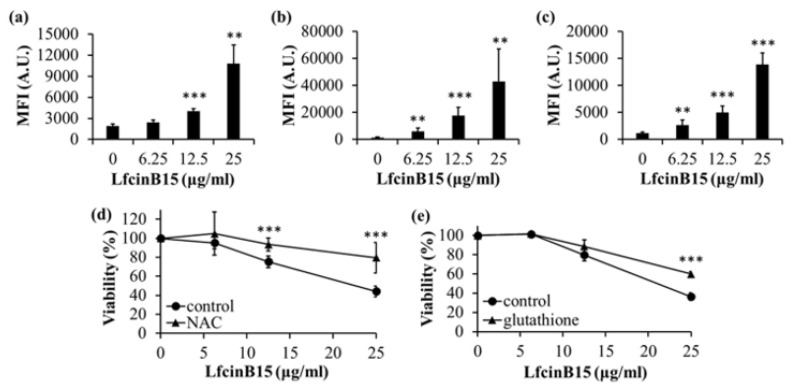
LfcinB15 induces ROS accumulation, and ROS levels correlate with the candidacidal activity of LfcinB15. ROS production was assessed by cell staining with DHE (**a**), H_2_DCFDA (**b**), and MitoSOX Red (**c**). The fluorescence intensity of the cells was analyzed using a flow cytometer. MFI, mean fluorescence intensity; A.U., arbitrary units. ** *p* < 0.01; *** *p* < 0.001. The effect of ROS scavengers on the candidacidal activity of LfcinB15 was also examined. Cells were pretreated with NAC (**d**) or glutathione (**e**), followed by incubation with various concentrations of LfcinB15 as indicated. The data are presented as the mean ± SD of three independent experiments. *** *p* < 0.001.

In addition to disturbance of membrane integrity, the induction of cellular ROS generation is another common mechanism for AMPs against *C. albicans* [37,38,39]. Remarkably, an abnormal surface appearance with protuberances was observed in LfcinB15-treated cells (Figure 1d), and this phenotype was correlated with the generation of ROS in *C. albicans* cells exposed to the antifungal miconazole and other AMPs [33,40,41]. Therefore, we further determined whether LfcinB15 can induce ROS generation. Cells were stained with the ROS indicators DHE and H_2_DCFDA, and the intracellular ROS level was measured using flow cytometry. As shown in Figure 4a,b, the mean fluorescence intensity (MFI) of cells treated with LfcinB15 was significantly increased compared to that of the untreated control. Moreover, the level of mitochondrial ROS was measured using the mitochondrial superoxide indicator MitoSOX Red. LfcinB15-treated cells had a higher level of MitoSOX Red fluorescence than the control cells (Figure 4c).

Excessive ROS production leads to oxidative stress, damages vital components of cells and causes cell death [42,43]. Because LfcinB15 induced ROS generation (Figure 4a–c), the ROS scavengers NAC and glutathione (GSH) were used to correlate ROS with the candidacidal activity of LfcinB15. NAC functions as an antioxidant by increasing the intracellular GSH levels and by directly reacting with ROS for detoxification [44,45]. *C. albicans* cells were pretreated with or without NAC, followed by incubation with various concentrations of LfcinB15, and the number of CFUs was counted. Pretreatment with NAC increased *C. albicans* viability compared to that of the control without NAC treatment (Figure 4d). This result indicated that NAC can rescue cells from LfcinB15 killing. Furthermore, GSH itself is an important antioxidant, reacting with free thiol groups in biological systems to protect cells from the harmful effects of ROS. Figure 4e shows that LfcinB15-induced cell death can also be rescued in cells pretreated with GSH. Our results suggested that LfcinB15 induced intracellular ROS production, which is likely one of the mechanisms for the candidacidal activity of LfcinB15.

### 3.5. The Hog1 and Mkc1 Mitogen-Activated Protein Kinases (MAPKs) Are Activated in Response to LfcinB15

*C. albicans* Hog1 is a stress-activated kinase that participates in the cell response to osmotic and oxidative stresses [46]. Interestingly, the *C. albicans hog1*Δ mutant is sensitive to the AMPs Hst 5 and human beta-defensins (hBD-2 and hBD-3), suggesting that Hog1 plays a role in the cell response to AMPs [47,48].

**Figure 5:**
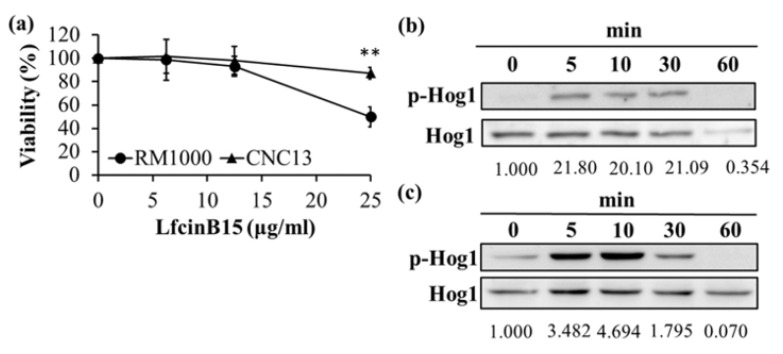
Hog1 is activated in *C. albicans* treated with LfcinB15. (**a**) Cells were treated with or without various concentrations of LfcinB, and cell viability is reported as a percentage. RM1000: *HOG1/HOG1* [49]; CNC13: *hog1*Δ*/hog1*Δ [50]. The data are presented as the mean ± SD of three independent experiments. ** *p* < 0.01. Hog1 activation induced by either 12.5 μg/mL (**b**) or 25 μg/mL (**c**) LfcinB15 was detected by western blotting and analyzed with ImageJ software. The total Hog1 band of each sample served as the loading control and was used to normalize the phosphor-Hog1 levels indicated by the fold-change values. The data are representative of three independent experiments with identical results.

Because LfcinB15 induced ROS production that triggered oxidative stress (Figure 4a–e), we also determined the possible involvement of Hog1 in the *C. albicans* response to LfcinB15. The results showed that the *hog1*Δ mutant was more tolerant to LfcinB15 than its wild-type parental strain (Figure 5a). Moreover, the phosphorylation levels of Hog1 were increased, particularly in cells treated with either 12.5 or 25 μg/mL LfcinB15 for 5, 10 and 30 min (Figure 5b,c). As Hog1 appeared to be involved in the cell response to LfcinB15, two other MAPKs, Mkc1 and Cek1, were also examined. Interestingly, activation of Mkc1, but not Cek1, was also detected (Figure S1 in the Supplementary Materials). Together, LfcinB15 treatment induces Hog1 and Mkc1 phosphorylation, suggesting that these MAPK pathways are involved in cell response against LfcinB15.

### 3.6. LfcinB15 Also Causes Mitochondrial Dysfunction

Mitochondria are the major site for ATP synthesis through oxidative phosphorylation that is linked to the electron transport chain (ETC) located in the inner mitochondrial membrane. Additionally, mitochondria are the major source of intracellular ROS that are mainly generated at complexes I and III of the ETC [51]. Because ROS generation is related to the *C. albicans*- killing activity of LfcinB15 (Figure 4a–e), we further investigated the possible effects of LfcinB15 on mitochondria by measuring the hallmarks of mitochondrial functions, including the mitochondrial membrane potential, the intracellular ATP level and the oxygen consumption rate (OCR) of cells.

**Figure 6:**
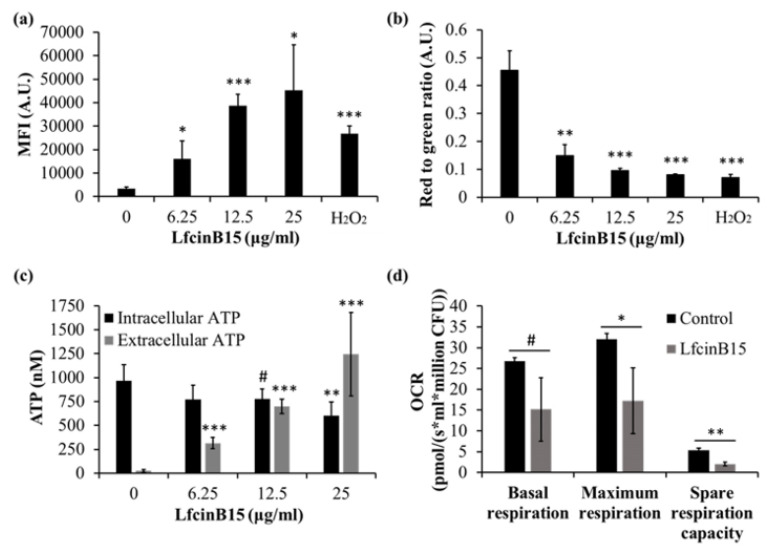
LfcinB15 affects mitochondrial functions. Measurement of mitochondrial membrane potential in cells treated with or without LfcinB15 by rhodamine 123 (**a**) and JC-1 (**b**) staining. The results are presented as the mean ± SD of three independent experiments. * *p* < 0.05, ** *p* < 0.01, *** *p* < 0.001. MFI, mean fluorescence intensity; A.U., arbitrary units. (**c**) The intracellular and extracellular ATP level of each sample was calculated from an ATP standard curve. The results are presented as the mean ± SD of at least three independent experiments. # *p* = 0.06, ** *p* < 0.01, *** *p* < 0.001. (**d**) The oxygen consumption rate (OCR) of cells treated with or without a sublethal dose of LfcinB15 (12.5 μg/mL) was measured with high-resolution respirometry using an Oroboros O2k system. The OCR was defined as pmol oxygen consumed per second per ml of the sample, and the OCR of each sample was normalized to the number of CFUs. The results are presented as the mean ± SD of three independent experiments. # *p* = 0.06, * *p* < 0.05, ** *p* < 0.01.

The mitochondrial membrane potential (ΔΨm) is an important indicator of mitochondrial activity, and a loss of ΔΨm represents mitochondrial dysfunction [52]. For the measurement of ΔΨm changes, two fluorescent dyes, rhodamine 123 and JC-1, were used. *C. albicans* cells were treated with and without LfcinB15 and stained with rhodamine 123 and JC-1. Simultaneously, cells treated with H_2_O_2_ were used as positive controls. When the mitochondrial membrane is depolarized, the fluorescence intensity of rhodamine 123 is expected to be increased, while the wavelength of JC-1 fluorescence will shift from red (maximum at 590 nm) to green (maximum at 530 nm). As shown in Figure 6a, the MFI of rhodamine 123 was largely increased in cells treated with LfcinB15 or H_2_O_2_ compared to untreated control cells. Moreover, the red-to-green fluorescence ratio of JC-1 was largely decreased in cells treated with LfcinB15 and H_2_O_2_ compared to untreated cells (Figure 6b).

The intracellular ATP level is tightly controlled to obtain a balance between ATP production and expenditure. Because bioenergetics is a predominant function of mitochondria, a reduction in ATP synthesis is another characteristic of mitochondrial dysfunction. Therefore, *C. albicans* cells were treated with and without LfcinB15, and the intracellular ATP level was assessed using a luciferin-luciferase assay. As expected, a decrease in the intracellular ATP level was observed in cells treated with various concentrations of LfcinB15 (Figure 6c). However, because LfcinB15 has membrane-disturbing activity (Figure 3), ATP may be leaked out of cells, and this leakage may affect the measurement of the intracellular ATP level. Therefore, in addition to the ATP levels within the cells, the extracellular ATP levels were determined. As shown in Figure 6c, the extracellular ATP levels were increased.

In parallel to measuring the mitochondrial membrane potential and ATP synthesis, mitochondrial respiration was also assessed in LfcinB15-exposed cells by measuring the OCR. Cells were treated with or without a sublethal dose of LfcinB15 (12.5 μg/mL) and analyzed with high-resolution respirometry using an Oroboros O2k system. After measuring the basal respiration, CCCP titration was conducted to detect the maximum respiration [53]. The spare respiration capacity was then calculated by subtracting basal respiration from maximal respiration [54]. Figure 6d shows that compared to the untreated control cells, cells treated with LfcinB15 showed a reduced OCR. Moreover, the maximum respiration and spare respiration capacity were reduced, suggesting the ability of LfcinB15 to suppress aerobic respiration. Together, our findings suggested that LfcinB15 treatment leads to mitochondrial dysfunction in *C. albicans* cells.

### 3.7. The Effect of LfcinB15 on Mitochondria Is Associated with the Candidacidal Activity of the Peptide

To continue to correlate the effect of LfcinB15 on mitochondria in terms of candidacidal activity, various *C. albicans* mutants that are deficient in mitochondrial ETC and ETC inhibitors were used.

**Figure 7:**
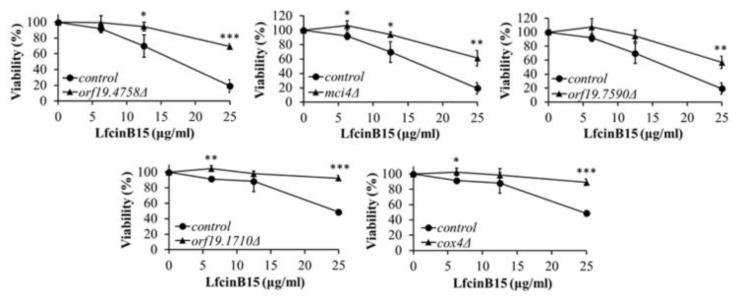
The susceptibility of *C. albicans* mutants that are deficient in mitochondrial ETC genes to LfcinB15. The viability of *C. albicans* mutants that are deficient in ETC genes (*orf19.4758*, *mci4*, *orf19.7590*, *orf19.1710*, and *cox4*) was compared to that of the parental SN250 strain. The results are presented as the mean ± SD of three independent experiments. * *p* < 0.05, ** *p* < 0.01, *** *p* < 0.001.

The LfcinB15 susceptibility of five mutants lacking mitochondrial complex I and IV genes (*orf19.4758*, *orf19.7590*, *mci4*, *orf19.1710*, and *cox4*) [23] was tested. Orf19.4758 is a putative reductase or dehydrogenase associated with mitochondrial complex I assembly [55], while Orf19.7590 is a putative NADH-ubiquinone oxidoreductase that is one of the mitochondrial complex I core subunits [55]. Moreover, Mci4 is a putative NADH-ubiquinone dehydrogenase and is also related to mitochondrial complex I assembly [55]. Orf19.1710 is also a putative NADH-ubiquinone oxidoreductase related to complex I activity, and Cox4 is a putative cytochrome c oxidase subunit [55]. The results showed that the control SN250 cells were sensitive to LfcinB15, whereas all the mutant strains were resistant to LfcinB15 (Figure 7).

**Figure 8:**
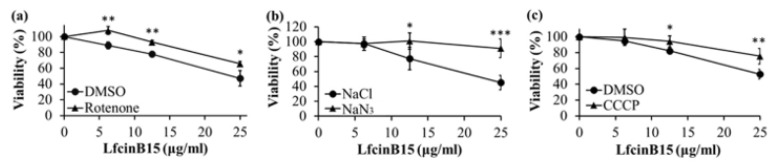
LfcinB15-mediated mitochondrial dysfunction is associated with *C. albicans* killing. The viability of cells cotreated with LfcinB15 and rotenone (**a**), NaN_3_ (**b**) or CCCP (**c**) was compared to that of the control as indicated. The results are presented as the mean ± SD of three independent experiments. * *p* < 0.05, ** *p* < 0.01, *** *p* < 0.001.

Moreover, rotenone is a mitochondrial complex I inhibitor that blocks NADH oxidation, which is the initial step of the ETC. *C. albicans* cells were treated with rotenone or DMSO (as a negative control), followed by treatment with various concentrations of LfcinB15. As shown in Figure 8a, cells treated with rotenone and LfcinB15 had higher viability than the control cells treated with DMSO and LfcinB15. In addition, NaN_3_ is an inhibitor of mitochondrial complex IV, and CCCP uncouples the mitochondrial ΔΨm with the ETC, both inhibiting ATP production. The results showed that cells cotreated with NaN_3_ and LfcinB15, or with CCCP and LfcinB15, had higher viability than the control cotreated with NaCl and LfcinB15, or with DMSO and LfcinB15, respectively (Figure 8b,c).

## 4. Discussion

LfcinB15 is an AMP derived from bovine lactoferricin B (LfcinB) and has even higher antibacterial activity than human lactoferricins [18]. However, the antifungal activity and detailed mechanism of LfcinB15 are still mostly unknown. Here, we therefore investigated the mechanisms of action by which LfcinB15 kills *C. albicans*.

In this study, LfcinB15 exhibited inhibitory activity against planktonic cells, biofilm cells, and clinical isolates of *C. albicans* and non-*albicans Candida* species (Figure 1a–c and Table 1). Nevertheless, we also determined the localization of LfcinB15 within *C. albicans* cells. LfcinB15 is observed not only to associate with the cell surface but also to enter cells and mainly accumulate in vacuoles (Figure 2). Moreover, LfcinB15 exhibits membrane lytic activity (Figure 3). Interestingly, an early study indicated that LfcinB, the parental peptide of LfcinB15, kills *C. albicans* cells without changing cell wall stability but does induce the release of potassium ions [34]. Hence, similar to that of LfcinB, our findings suggest that disturbing membrane integrity is one of the mechanisms related to the inhibitory activity of LfcinB15.

Redox homeostasis is vital for *C. albicans* survival. The impacts of AMPs on mitochondrial redox balance to induce ROS accumulation have been reported in several studies [38,56,57]. Increased ROS levels lead to oxidative stress, and ROS can induce cell death. In this study, LfcinB15 induced ROS generation that may cause oxidative stress, and ROS accumulation was associated with the killing activity of LfcinB15 (Figure 1d and Figure 4a–e). Furthermore, mitochondria are considered a major source of ROS [42], and mitochondrial dysfunction is often associated with the accumulation of ROS [58]. Indeed, several antifungal agents and AMPs that induce ROS generation can also cause mitochondrial dysfunction. For example, both kalopanaxsaponin A (KPA) and the AMP hepcidin 25 induce ROS generation in *C. albicans* [56,59]. Additionally, KPA causes a significant decrease in the intracellular ATP level in a dose-dependent manner [59], and hepcidin 25 is able to reduce the mitochondrial membrane potential [56]. Because LfcinB15 induces ROS generation, several hallmarks of mitochondrial dysfunction were thus examined. In Figure 6a–d, depolarization of the mitochondrial membrane, decreased intracellular ATP content and reduced OCR are shown in cells treated with LfcinB15. Mitochondrial ROS generation mainly occurred through the production of superoxide from complexes I and III of the ETC, although other sites can also contribute. Importantly, mitochondrial dysfunction appears to be associated with the *C. albicans*-killing activity of LfcinB15, as further indicated by different ETC inhibitors including rotenone, NaN3 and CCCP, and mitochondrial-deficient mutants (Figure 7). Furthermore, it is interesting to note that cells cotreated with the ETC inhibitors and LfcinB15 had higher viability than the controls (Figure 8a–c). Because ETC generates a proton gradient that drives ATP synthesis (in complex V) and ROS generation occurs in the ETC, all the inhibitors blocking ETC can lead to reduced ROS generation and ATP synthesis. Interestingly, Helmerhorst et al. also demonstrated that cells cotreated with NaN_3_ or cyanide can rescue the killing by Hst 5, and concluded that Hst 5 targets the energized mitochondria [60]. Together, our results suggest that ROS, oxidative stress and mitochondrial dysfunction are also related to *C. albicans* killing by LfcinB15, apart from membrane disturbance.

In addition to the change of intracellular ATP levels, LfcinB15 is able to induce the release of ATP into the extracellular environment (Figure 6c). Interestingly, released cellular ATP is associated with candidacidal activity by some cationic AMPs and regulation of cellular functions in higher eukaryotes [11,61]. For example, the addition of apyrase, an ATP scavenger, can reduce Hst 5-mediated killing, suggesting a direct connection between released ATP and *C. albicans* cell death [62]. Moreover, released ATP modulates functions of immune cells through its activation of purinergic receptors [63]. Although the underlying mechanisms are not fully understood, treatment with purinergic antagonists prevents Hst 5-mediated *C. albicans* killing [64]. Finally, the release of ATP induced by Hst 5 occurs without membrane lysis and is mediated by ion transporters [61]. However, our results indicated that LfcinB15 is able to disturb membrane integrity and also induce ATP release (Figure 6c). Therefore, several questions still need to be addressed to discern different mechanisms between LfcinB15 and Hst 5. For example, how does LfcinB15 interact with *C. albicans* cells? Is recognition and binding with cell surface proteins or other components required for the candidacidal activity of LfcinB15? Finally, is released ATP involved in LfcinB15-mediated cell death?

Moreover, LfcinB15 treatment induced Hog1 activation, but unexpectedly, the *hog1*Δ mutant was more resistant to LfcinB15 (Figure 5a–c). Interestingly, similar findings have been reported for other fungal pathogens treated with different antifungal reagents. For example, the *OSC1* gene encodes an ortholog of Hog1 in the plant pathogen *Colletorichum lagenarium*. Treatment with fludioxonil activates Osc1 phosphorylation, whereas the *osc1*Δ mutant was more resistant to fludioxonil [65]. Moreover, *C. albicans* Nik1 is a two-component histidine kinase functioning upstream of Hog1 [66,67,68]. Using a *Saccharomyces cerevisiae* strain (named CaNik1) with heterologous expression of *C. albicans* Nik1, Hog1 is activated upon treatment with fludioxonil [66]. The CaNik1 strain with *HOG1*-deletion is also resistant to fludioxonil [66]. Finally, other than Hog1, LfcinB15 can also induce the activation of Mkc1 (Figure S1 in the Supplementary Materials). Although the LfcinB15 resistance of the *hog1*Δ mutant and possible interplay between Hog1 and Mkc1 signaling pathways remain elusive, our study shows the complexity in cell responses to AMPs.

Of special interest in the present study is that LfcinB15 intracellularly accumulates in vacuoles, whereas mitochondrial functions are affected. Notably, similar phenomena were previously reported from studies of Hst 5 [11,57]. These observations raise an interesting question regarding how LfcinB15 functions between mitochondria and vacuoles. Recently, a molecular hub connecting the mitochondria and vacuole was identified in baker’s yeast *S. cerevisiae* [69,70]. Specifically, this hub is called the vacuole and mitochondria patch (vCLAMP) and consists of several proteins including Mcp1, Vam6, and the small GTPase Ypt7 [71]. However, there is only very limited information about the vCLAMP in *C. albicans*. Vam6 is involved in the maintenance of mitochondrial and vacuolar functions under oxidative stress in *C. albicans* [72]. In addition, Mcp1 contributes not only to mitochondrial maintenance, but also to mitophagy [73]. Because LfcinB15 treatment is likely to induce oxidative stress and causes mitochondrial dysfunction, it is possible that LfcinB15 killing is associated with the vCLAMP. Ypt72 is the *C. albicans* homolog of *S. cerevisiae* Ypt7 (http://www.candidagenome.org, accessed on 17 May 2021). Intriguingly, we found that the *C. albicans ypt72*Δ mutant was more tolerant to LfcinB15 than its wild-type parental strain (Figure S2 in the Supplementary Materials). However, the details underlying this finding for the possible relationship between LfcinB15 and the vCLAMP must be further investigated.

Considering the potential future use of LfcinB15, we have also initiated an investigation to determine the efficacy of LfcinB15 against different *Candida* species. LfcinB15 is a broad-spectrum AMP that deploys its activity against different clinical isolates of *Candida* species (Table 1). Importantly, many of these isolates are fluconazole resistant. Moreover, two *C. glabrata* isolates were much more resistant to LfcinB15 than *C. albicans* and other tested isolates. Similarly, resistance of *C. glabrata* to other AMPs such as *p*-113, VLL-28 and some other LfcinB-derived peptides was also reported [33,34,35]. In terms of mitochondrial structure and function, several possible reasons may be used to explain the different LfcinB15 susceptibility between *C. glabrata* and *C. albicans*. The mitochondrial ETC of *C. glabrata* resembles that of *S. cerevisiae* rather than *C. albicans*, and does not express a multi-subunit complex I [74]. In addition, the OCR of *C. glabrata* cells is significantly lower than that of *C. albicans* [75]. However, clarifying the different LfcinB15 susceptibility will require more clinical isolates of *C. glabrata* for future study.

## 5. Conclusions

In summary, we demonstrated that LfcinB15 exhibits anti-*Candida* activity against planktonic cells, biofilm cells and clinical isolates. Moreover, LfcinB15 possesses multiple modes of action in *C. albicans* killing, including causing mitochondrial damage. Recently, much attention has been paid to exploring mitochondria as new targets for the development of antifungals [76,77]. Here, our findings of LfcinB15 imply that communication between organelles may also be involved in AMP-mediated killing, although the detailed mechanisms remain to be elucidated. Overall, our results, in combination with others, highlight the complex mechanisms that may be used by AMPs and suggest the potential use of AMPs in therapeutic applications for infectious diseases.

## Figures and Tables

**Table 1:** Antifungal activity of LfcinB15 against clinical isolates of *Candida* spp.

*Candida* spp.	Source ^a^	Strains	MIC (μg/mL)
*C. albicans*	ATCC MYA-2876	SC5314	6.25
*C. albicans*	ATCC90028	YLO12	6.25
*C. albicans*	HIV patient	YH050001	6.25
*C. albicans*	HIV patient	YH050005	12.5
*C. albicans*	HIV patient	YH050072 ^b^	6.25
*C. glabrata*	ATCC9003	YLO8	>50
*C. glabrata*	HIV patient	YH050105	50
*C. krusei*	ATCC6258	YLO6	12.5
*C. krusei*	HIV patient	YH050075 ^b^	6.25
*C. tropicalis*	ATCC13803	YLO86 ^b^	3.125
*C. tropicalis*	HIV patient	YH050007 ^b^	3.125
*C. tropicalis*	HIV patient	YH050013 ^b^	6.25
*C. tropicalis*	HIV patient	YH050114 ^b^	6.25
*C. parapsilosis*	ATCC22019	YLO7	3.125
*C. dubliniensis*	HIV patient	YH050092	12.5

^a^ HIV patient, *Candida* strains isolated from HIV-infected patients. ^b^ Fluconazole-resistant isolates.

## Data Availability

The data presented in this study are available within the article and the Supplementary Materials.

## Supplementary Materials

The following are available online at https://www.mdpi.com/article/10.3390/jof7070519/s1. Figure S1: LfcinB15 treatment activates C. albicans Mkc1. Figure S2: The C. albicans ypt72-deleted mutant is more tolerant to LfcinB15.
