# Supplementary material for: Antimicrobial Activity of the Peptide LfcinB15 against Candida albicans
Source: J Fungi (Basel). 2021 Jun 29;7(7):519. doi: 10.3390/jof7070519 (PMC8306953; doi:10.3390/jof7070519)
Supplement: Supplementary file 1 [file jof-07-00519-s001.zip › jof-1243612-supplementary.pdf]

Supplementary material

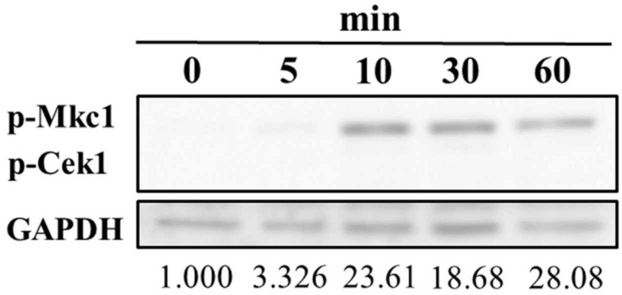

Figure S1 LfcinB15 treatment activates *C. albicans* Mkc1. Activation of Mkc1 but not Cek1 induced by 12.5 μg/ml LfcinB15 was detected by western blotting and analyzed with ImageJ software. The GAPDH band of each sample served as the loading control and was used to normalize the phosphorylated Mkc1 levels indicated by the fold change values. The data are representative of three independent experiments with identical results.

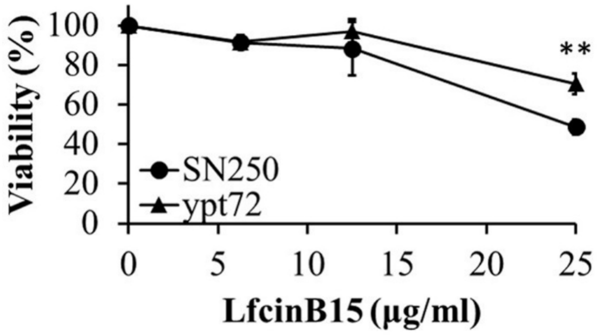

Figure S2 The *C. albicans ypt72*-deleted mutant is more tolerant to LfcinB15. The viability of *ypt72*Δ mutant cells treated with LfcinB15 was compared to that of the parental SN250 strain. The results are presented as the mean ± SD of three independent experiments. **p<0.01.
